# Autoimmune Hepatitis Mimicking Pancreatico-Biliary Malignancy

**DOI:** 10.7759/cureus.114708

**Published:** 2026-08-18

**Authors:** Harsh Anadkat, Nakul Morakhia

**Affiliations:** 1 Medicine, Sir Sayajirao General Hospital Medical College Baroda, Vadodara, IND; 2 Gastroenterology, Morakhia Heart and Gastro-Liver Hospital, Gandhidham, IND

**Keywords:** antinuclear antibody, autoimmune hepatitis (aih), azathioprine therapy, diagnostic mimicry, elevated ca 19-9, hepatocellular jaundice, pancreatic head mass, pancreatic head mass mimic, very elderly patient

## Abstract

A man in his 80s presented with a one-week history of jaundice, abdominal pain, low-grade fever, pruritus, and anorexia. Biochemistry showed a hepatocellular pattern of liver injury, with aspartate aminotransferase of 867 U/L and alanine aminotransferase of 702 U/L, alongside conjugated hyperbilirubinemia and a prolonged international normalized ratio. Contrast-enhanced computed tomography of the abdomen showed a bulky pancreatic head and uncinate process with mild common bile duct dilatation and no discrete mass, alongside a markedly elevated CA 19-9 of 225 U/mL, which together raised initial concern for pancreatico-biliary malignancy. A comprehensive viral hepatitis panel, including hepatitis A, B, C, and E, and thyroid function testing were unremarkable. Further workup revealed a strongly positive antinuclear antibody at high titer and an elevated total immunoglobulin G. A clinical diagnosis of autoimmune hepatitis was made using simplified international diagnostic criteria; liver biopsy was offered but declined by the patient due to his advanced age and practical constraints. Treatment with corticosteroids and azathioprine led to complete biochemical normalization over several weeks, with sustained improvement on maintenance therapy. This case demonstrates that autoimmune hepatitis can closely mimic pancreatico-biliary malignancy on cross-sectional imaging and tumor markers in an older patient, and highlights the diagnostic value of autoimmune serology and immunoglobulin G levels in avoiding unnecessary invasive oncological investigation.

## Introduction

Autoimmune hepatitis is a chronic, immune-mediated liver disease of unknown cause, and its incidence and prevalence have been rising globally over recent decades [1]. It classically affects younger women, but it can present at any age, including in older men [2,3]. In this group, it is often overshadowed by more common explanations for jaundice, such as malignant biliary obstruction, and diagnosis can be challenging even when typical features are present [4]. When cross-sectional imaging shows an ambiguous pancreatic finding alongside jaundice, clinicians may be drawn toward an oncological workup before an autoimmune cause is considered. This case is presented because it shows how a bulky, non-mass-forming pancreatic head, together with a markedly elevated CA 19-9, can closely mimic pancreatico-biliary malignancy. The true diagnosis here was reached through a hepatocellular biochemical pattern, autoimmune serology, and immunoglobulin G levels, which avoided an unnecessary invasive oncological pathway.

## Case presentation

A man in his 80s presented with a one-week history of jaundice and abdominal pain, associated with low-grade fever, pruritus, and anorexia. He had no past history of diabetes mellitus, hypertension, chronic liver disease, or regular drug intake, and no history of alcohol use or a relevant family history of liver or autoimmune disease. On examination, he had icterus and mild pedal edema. A cardiology opinion was sought for the edema; low-dose diuretics and a low-salt diet were advised, and no cardiac dysfunction was found to explain it. He had no ascites, hepatomegaly, or splenomegaly, and no other stigmata of chronic liver disease.

**Figure 1:**
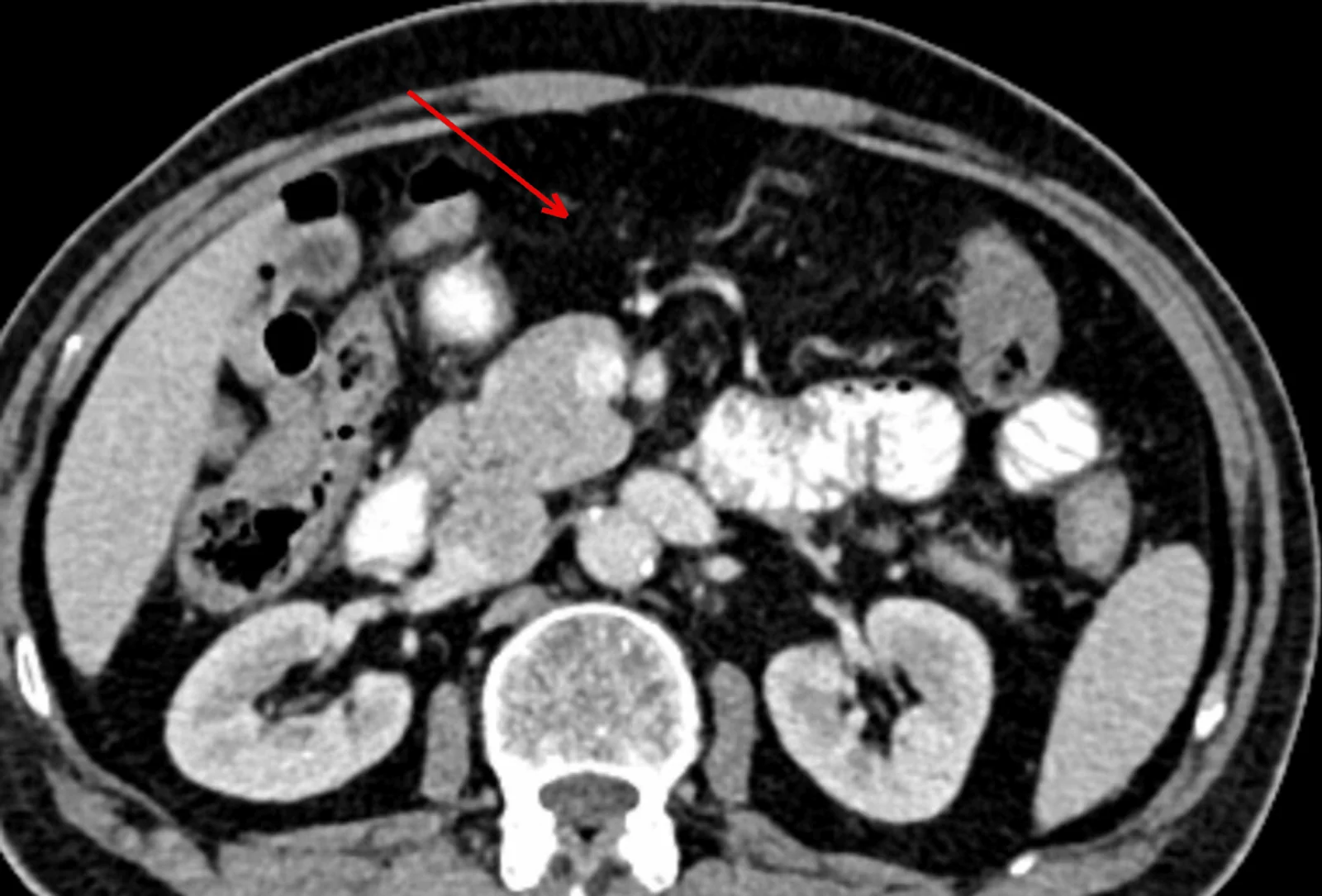
Venous-phase contrast-enhanced abdominal CT demonstrating a bulky pancreatic head and uncinate process (arrow) with a patent portal venous system

**Figure 2:**
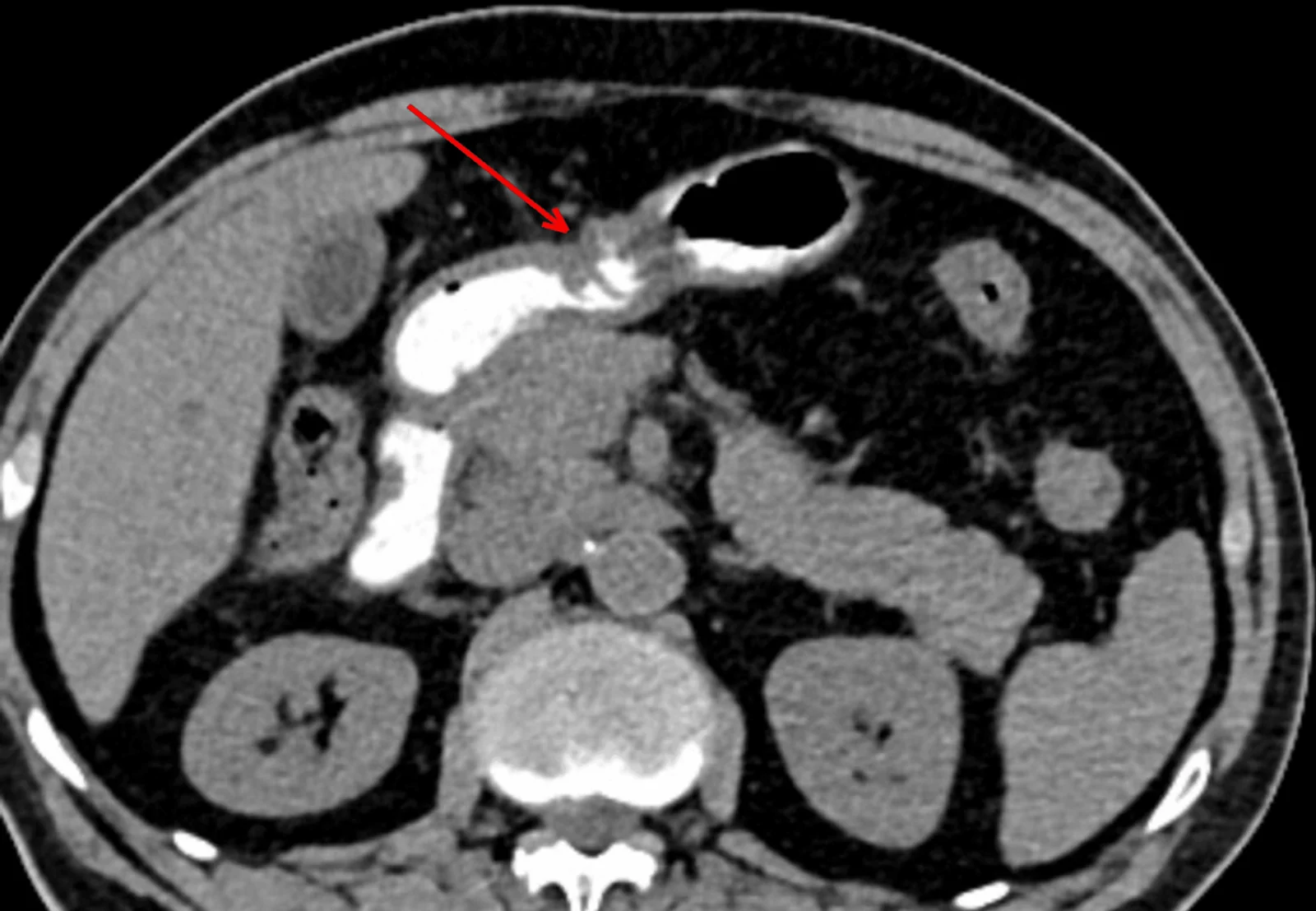
Plain-phase (non-contrast) CT of the abdomen showing a pancreatic head and uncinate process (arrow)

**Figure 3:**
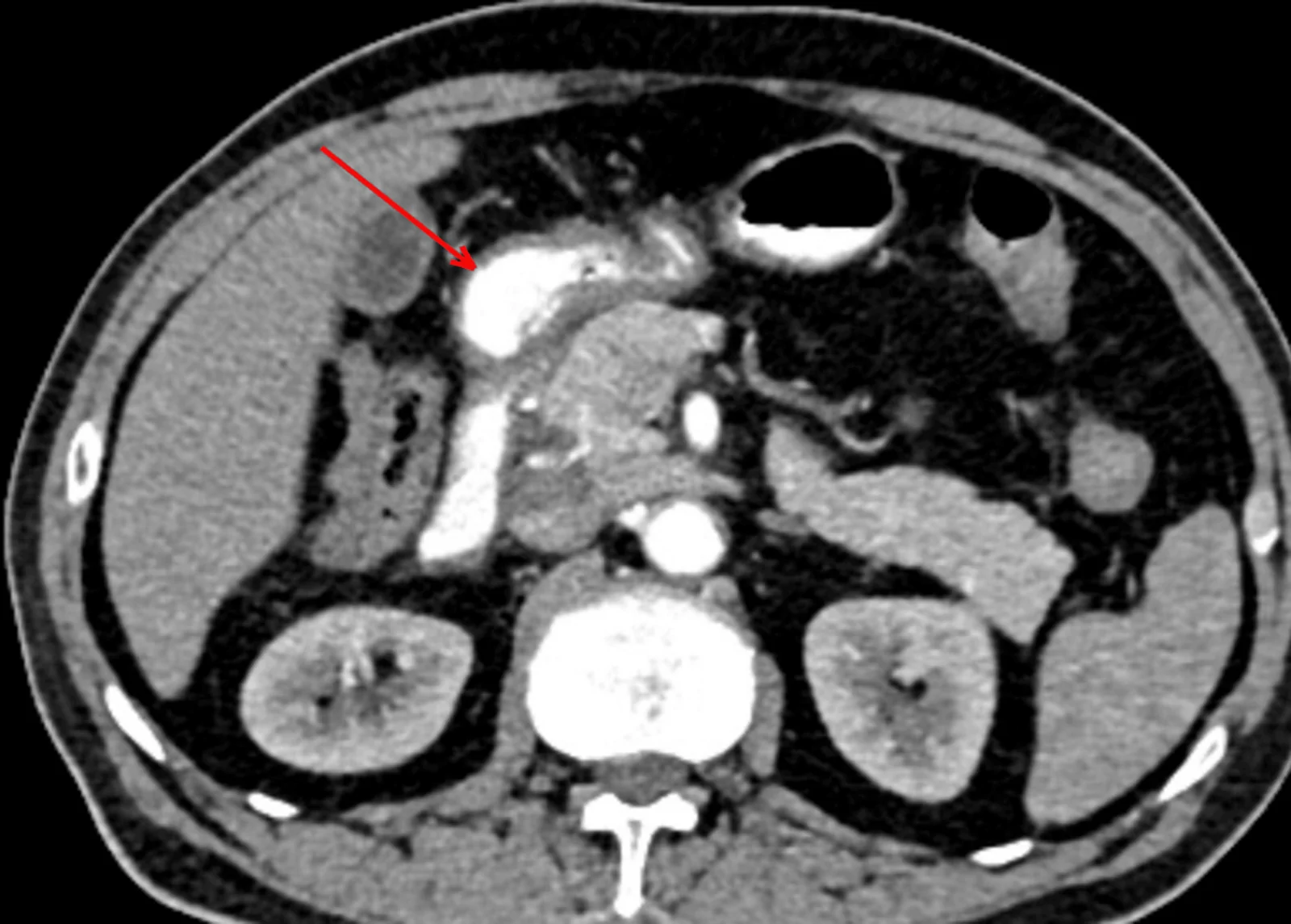
Arterial-phase contrast-enhanced abdominal CT demonstrating a bulky pancreatic head and uncinate process (arrow) without a discrete mass lesion

Initial biochemistry showed a hepatocellular pattern of liver injury with conjugated hyperbilirubinemia: total bilirubin 8.90 mg/dL, direct bilirubin 7.03 mg/dL, aspartate aminotransferase 867.4 U/L, alanine aminotransferase 702.5 U/L, alkaline phosphatase 150 U/L, and an international normalized ratio of 1.5. CA 19-9 was markedly elevated at 225 U/mL (Table 1). Anti-hepatitis A IgM, hepatitis B surface antigen, anti-hepatitis C, and anti-hepatitis E IgM were all negative, and thyroid function was normal. Contrast-enhanced CT of the abdomen showed a bulky pancreatic head and uncinate process with maintained peripancreatic fat planes and a non-dilated pancreatic duct, and no discrete mass. There was mild common bile duct dilatation with minimal central intrahepatic biliary radicle dilatation, and mild gallbladder wall thickening without calculus. No lymphadenopathy or free fluid was seen (Figures 1-3).

**Table 1:** Initial laboratory investigations at presentation, showing a hepatocellular pattern of liver injury with conjugated hyperbilirubinemia and only mild elevation of alkaline phosphatase, alongside a markedly elevated CA 19-9

Parameter	Value	Reference range
Total bilirubin (mg/dL)	8.90	0.2-1.2
Direct bilirubin (mg/dL)	7.03	0.0-0.5
Aspartate aminotransferase (AST) (U/L)	867.4	0-35
Alanine aminotransferase (ALT) (U/L)	702.5	0-45
Alkaline phosphatase (U/L)	150	40-130
International normalized ratio (INR)	1.5	0.8-1.1
CA 19-9 (U/mL)	225	0-39

This combination of findings initially raised concern for pancreatico-biliary malignancy. However, the absence of a discrete pancreatic mass, a non-dilated pancreatic duct, and the lack of lymphadenopathy argued against this, as did the disproportionately hepatocellular pattern of liver injury, with alkaline phosphatase only mildly raised relative to the striking transaminase elevation. Given this, autoimmune serology was checked: antinuclear antibody by immunofluorescence was strongly positive (speckled pattern, titer 1:1000), and total immunoglobulin G was elevated at 2065 mg/dL (reference 700-1600 mg/dL). Using the simplified International Autoimmune Hepatitis Group scoring criteria, the presentation was consistent with probable autoimmune hepatitis (Table 2).

**Table 2:** Simplified International Autoimmune Hepatitis Group (IAIHG) diagnostic score Interpretation: score ≥ 7 = definite autoimmune hepatitis; score 6 = probable autoimmune hepatitis. The patient scored 6, consistent with probable autoimmune hepatitis.

Parameter	Patient's finding	Points awarded	Maximum possible
ANA or SMA titer	ANA 1:1000 (≥1:80)	2	2
IgG level	2065 mg/dL (>1.1 × ULN of 1600)	2	2
Liver histology	Not performed (biopsy declined)	0	2
Exclusion of viral hepatitis	Hepatitis A, B, C, and E all negative	2	2
Total score		6	8

Liver biopsy was offered but declined by the patient, largely because of his advanced age and the practical challenges of an invasive procedure and follow-up while living at a distance from tertiary care. A clinical diagnosis of autoimmune hepatitis was therefore made. Treatment began with oral prednisolone 15 mg daily, continued for 20 days, then tapered to 10 mg daily, and after a further 10 days tapered to 5 mg daily. Azathioprine 50 mg daily was added approximately two weeks into the treatment; complete blood counts were checked twice over the following two weeks to screen for cytopenia, both of which were normal, after which azathioprine was increased to 75 mg daily. Calcium and vitamin D supplementation were started for osteoporosis prophylaxis.

Serial biochemistry showed progressive improvement over the follow-up period (Table 3). By final follow-up, bilirubin and aspartate aminotransferase had normalized completely, and the initially prolonged international normalized ratio had improved, reflecting recovery of synthetic liver function alongside resolution of hepatocellular injury. The plan going forward is to continue maintenance therapy with prednisolone 5 mg daily and azathioprine 75 mg daily for a minimum of two years to prevent relapse, with liver function monitored every two months. At the time of writing, the patient remains well, with normal liver function, and has returned to his usual daily activities.

**Table 3:** Serial biochemistry during treatment, showing progressive normalization of total bilirubin and aspartate aminotransferase, alongside improvement of the initially prolonged international normalized ratio, over the course of corticosteroid and azathioprine therapy

Timepoint	Total bilirubin (mg/dL)	AST (U/L)	INR
Presentation	8.9	867.4	1.5
1 week later	9.4	534	1.4
12 days later	6.14	220.2	1.3
36 days later	1.86	54.5	1.1
Final follow-up	1	25 (normal, <45)	1.1

## Discussion

This case makes two educational points. First, autoimmune hepatitis must remain in the differential diagnosis for hepatocellular-pattern jaundice in older men, even though it is classically considered a disease of younger women. Second, incidental or reactive imaging and tumor-marker findings can create a false trail toward malignancy. CA 19-9 is a nonspecific marker that rises with cholestasis and inflammatory conditions and can reach substantial levels without any underlying cancer [5]. The absence of a discrete pancreatic mass and a normal pancreatic duct caliber were key reassuring imaging features in this case. Liver biopsy remains the reference standard for confirming autoimmune hepatitis and excluding overlap syndromes [6]. Its omission here, declined by the patient because of his age and practical constraints, did not prevent a confident clinical diagnosis using validated simplified International Autoimmune Hepatitis Group criteria [7]. This diagnosis was further supported by a robust and sustained treatment response, with complete normalization of bilirubin, transaminases, and the international normalized ratio over several weeks of corticosteroid and azathioprine therapy.

## Conclusions

Autoimmune hepatitis can present in elderly men with a hepatocellular pattern of jaundice that closely mimics pancreatico-biliary malignancy on cross-sectional imaging, particularly when a bulky, non-mass-forming pancreatic head is accompanied by an elevated CA 19-9. Recognizing the disproportionately hepatocellular biochemical pattern, combined with autoimmune serology and immunoglobulin G levels, allowed a confident diagnosis using the simplified International Autoimmune Hepatitis Group criteria without the need for liver biopsy. Prompt initiation of corticosteroids and azathioprine led to complete biochemical normalization. This case highlights the importance of considering autoimmune hepatitis in the differential diagnosis of unexplained jaundice in older patients, even when imaging findings initially raise concern for malignancy, to avoid unnecessary invasive investigations and ensure timely, appropriate treatment.
